# Clinicopathological and prognostic significance of SWI/SNF complex subunits in undifferentiated gastric carcinoma

**DOI:** 10.1186/s12957-022-02847-0

**Published:** 2022-12-04

**Authors:** Zhenkun Zhang, Qiujing Li, Shanshan Sun, Zhe Li, Zheng Guo Cui, Menglan Zhang, Qian Liu, Yujie Zhang, Sili Xiong, Shukun Zhang

**Affiliations:** 1 Weihai Municipal Hospital, Shandong University, Weihai, 264200 Shandong China; 2 Department of Oncology, Shouguang People’s Hospital, Weifang, 262700 Shandong China; 3 Department of Pathology, Weihai Municipal Hospital, Shandong University, Weihai, 264200 Shandong China; 4 Department of Oncology, Weihai Municipal Hospital, Shandong University, Weihai, 264200 Shandong China; 5 Weifang Medical College, Weifang, 261053 Shandong China; 6 Department of Environmental Health, University of Fukui School of Medical Science, 23-3 Matsuoka Shimoaizuki, Eiheiji, Fukui 910-1193 Japan; 7 Department of Pathology, Qinghai Provincial People’s Hospital, Xining, 810000 Qinghai China

**Keywords:** SWI/SNF, SMARCA2, SMARCA4, Undifferentiated, Gastric carcinoma

## Abstract

**Background:**

The switch/sucrose nonfermentable (SWI/SNF) complex is an evolutionarily conserved chromatin remodeling complex that displays dysfunction in many tumors, especially undifferentiated carcinoma. Cancer stem cells (CSC), a special type of undifferentiated cancer cells with stem cell-like properties, play an essential role in tumor cell proliferation, invasion, and metastasis. In undifferentiated gastric carcinomas, the association of SWI/SNF complexes with clinicopathological features, CSC phenotype, and the prognosis is not fully understood.

**Methods:**

We collected a cohort of 21 patients with undifferentiated/dedifferentiated gastric carcinoma. We next performed immunohistochemistry staining for the five subunits of the SWI/SNF complex (ARID1A, ARID1B, SMARCA2, SMARCA4, and SMARCB1), and four mismatch repair proteins (MLH1, PMS2, MSH2, and MSH6), as well as other markers such as p53, PD-L1, and cancer stem cell (CSC) markers (SOX2, SALL4). Then, we investigated the correlation of SWI/SNF complex subunits with clinicopathological characters and performed prognostic analysis.

**Results:**

We observed SMARCA2 loss in 12 cases (57.14%), followed by ARID1A (5 cases, 23.81%) and SMARCA4 (3 cases, 14.29%). Fourteen cases (66.67%) lost any one of the SWI/SNF complex subunits, including 3 cases with SMARCA2 and ARID1A co-loss, and 3 cases with SMARCA2 and SMARCA4 co-loss. Correlation analysis revealed that the CSC phenotype occurred more frequently in the SWI/SNF complex deficient group (*P* = 0.0158). Survival analysis revealed that SWI/WNF complex deficiency, undifferentiated status, CSC phenotype, and the loss of SMARCA2 and SMARCA4 resulted in worse survival. Univariate and multivariate Cox regression analyses screened out three independent factors associated with worse prognosis: undifferentiated status, SWI/SNF complex deficiency, and lymph node metastasis.

**Conclusions:**

The SWI/SNF complex deficiency was more likely to result in a CSC phenotype and worse survival and was an independent prognostic factor in undifferentiated/dedifferentiated gastric carcinoma.

**Supplementary Information:**

The online version contains supplementary material available at 10.1186/s12957-022-02847-0.

## Background

Undifferentiated gastric carcinoma is a histopathologically rare, highly aggressive malignancy composed of cells showing no specific cytologic or architectural differentiation, including no gland formation or mucin production and no neuroendocrine, squamous, or sarcomatoid differentiation [1]. It is called dedifferentiated carcinoma if undifferentiated carcinoma contains a minor differentiated component. Pure undifferentiated carcinoma is often difficult to diagnose by morphology, and immunohistochemistry shows focal keratin or epithelial membrane antigen expression with no other cellular differentiation, which can be used to support its diagnosis. Meanwhile, due to its rarity, the clinical behavior of undifferentiated/dedifferentiated gastric carcinoma has not been thoroughly analyzed.

The switch/sucrose nonfermentable (SWI/SNF) complex is an evolutionarily conserved chromatin remodeling complex composed of more than 20 subunits, of which the five essential subunits are as follows: SWI/SNF-related, matrix-associated, actin-dependent regulator of chromatin, subfamily A, member (SMARCA4) and SMARCA2 are catalytic subunits that can catalyze adenosine triphosphatase (ATPase) to generate the energy required for chromatin remodeling. SMARCB1 is a core subunit associated with undifferentiated/rhabdoid carcinomas. AT-rich interaction domain 1A (ARID1A) and ARID1B are auxiliary regulatory subunits that form DNA binding domains [2]. SWI/SNF complex plays an important role in cellular processes, such as cell proliferation, lineage differentiation, and DNA repair [3]. It has been demonstrated that loss of the SWI/SNF complex subunits is associated with undifferentiated histological morphology [4], further leading to a more aggressive clinical behavior and worse outcome. Cancer stem cells (CSC) are a specialized subpopulation of undifferentiated tumor cells with stem cell-like properties that maintain tumor viability through self-renewal and unlimited proliferation [5], which can be identified by specific markers. In this study, we defined SRY-box transcription factor 2 (SOX2) or Spalt-like transcription factor 4 (SALL4) positivity as the CSC phenotype [6, 7], which is characterized by high self-renewal capacity, strong invasiveness, and resistance to chemotherapy and radiotherapy [5].

Many studies on the loss of SWI/SNF complex subunits in conventional gastric adenocarcinoma have been reported [8, 9]. Conversely, in undifferentiated/dedifferentiated gastric carcinoma, studies related to SWI/SNF complex are limited due to the rarity of cases. Herein, we investigated the immunohistochemical expression of five subunits (ARID1A, ARID1B, SMARCA2, SMARCA4, and SMARCB1) of the SWI/SNF complex in 21 undifferentiated/dedifferentiated gastric carcinomas, as well as other import immunohistochemical markers, including P53, programmed cell death ligand 1(PD-L1), SOX2, SALL4, and mismatch repair proteins MutL homolog 1 (MLH1), MutS homolog 2 (MSH2), MSH6, and PMS1 homolog, mismatch repair system component 2 (PMS2). We performed correlation analysis and prognostic analysis to reveal the correlation of SWI/SNF complex with clinicopathological features and prognosis.

## Methods

### Cases collection

We retrospectively screened 1271 patients with gastric adenocarcinoma treated by surgical resection at Weihai Municipal Hospital between January 2014 and December 2020. We then carefully reviewed the hematoxylin and eosin (H&E)-stained tissue sections to determine the undifferentiated and dedifferentiated morphologies and performed CK-pan staining to determine the epithelial origin. In addition, we performed the immunohistochemical staining of CD56, CgA, Syn, NSE, Vimentin, and Desmin, as well as the Epstein-Barr virus (EBV)-encoded small RNA in situ hybridization (EBER-ISH) to exclude neuroendocrine carcinoma, small cell carcinoma, sarcoma, carcinosarcoma, and EBV-positive gastric carcinoma. Finally, we identified 21 cases with undifferentiated/dedifferentiated gastric carcinoma, all of whom received 4–6 cycles of standard regimen chemotherapy after surgery. We obtained relevant information on patients’ demographics, tumor characteristics, and partial clinical outcomes from the electronic medical record system. We restaged the cases according to the current AJCC TNM staging system (8th edition, 2019). The median follow-up time was 35.20 months (range from 5.03 to 74.50 months). This study was approved by the Ethics Review Board of the Weihai Municipal Hospital (permission code: 2021053).

### Immunohistochemistry

We performed immunohistochemistry staining on representative slides derived from formalin-fixed and paraffin-embedded (FFPE) tissue blocks for each of the 21 cases using an automated immunostaining machine (Benchmark ULTRA, Ventana) according to the manufacturer's protocol and the instructions of the primary antibody. Details of primary antibodies are listed in Table 1.

**Table 1:** Summary of antibody information

Antibody	Clone	Source	Dilution	Manufacturer
ARID1A	EPR13501	Rabbit	1:1000	Abcam
ARID1B	2D2	Mouse	1:500	Abcam
SMARCA2	EPR23103-44	Rabbit	1:400	Abcam
SMARCA4	E8V5B	Rabbit	working solution	Origene
SMARCB1	25	Mouse	working solution	Origene
MSH2	RED2	Rabbit	working solution	Origene
MSH6	EP49	Rabbit	working solution	Origene
MLH1	ES05	Rabbit	working solution	Origene
PMS2	EP51	Rabbit	working solution	Origene
P53	DO-7	Mouse	working solution	Origene
PD-L1	SP263	Rabbit	working solution	Ventana, Roche
SOX2	EP103	Rabbit	working solution	Origene
SALL4	6E3	Mouse	working solution	Origene
CK-pan	AE1/AE3	Mouse	working solution	Origene

The five subunits of the SWI/SNF complex (ARID1A, ARID1B, SMARCA2, SMARCA4, and SMARCB1) were defined as “lost” if nuclear staining was completely absent, “reduced” if the staining intensity of > 90% tumor cells was significantly weaker than that of normal cells, and “intact” if the staining intensity of > 90% tumor cells was similar to that of normal cells [10]. We integrate the reduced and intact patterns into a present pattern according to previous studies [11]. As a positive control, intense homogeneous nuclear staining was seen in interstitial fibroblasts, inflammatory cells, vascular endothelial cells, or normal epithelial cells. Loss of any of the five SWI/SNF complex subunits was defined as deficient, and the existence of all five subunits was defined as intact. The four MMR proteins (MLH1, PMS2, MSH2, and MSH6) were all located in the nucleus and were assessed as present (unequivocal nuclear staining) or lost (complete absence of nuclear staining). Loss of any one of MMR proteins was defined as MMR-deficient (dMMR), and the presence of all four MMR proteins was defined as MMR-proficient (pMMR). P53 was defined as mutant when > 90% of tumor cell nuclei were homogeneously strongly stained or with a complete absence of staining [12]. SALL4 was defined as positive when any stained tumor cells were observed [7]. The positive criteria for PD-L1 [13] and SOX2 [6] were > 1% and > 10% of stained cells, respectively.

### Statistical analysis

The correlation between SWI/SNF complex status and categorical variables was tested by Fisher's exact test (*n* < 40), and the correlation between SWI/SNF complex status and continuous variables (i.e., age, size) was assessed by *t*-test (normal distribution) or Mann Whitney *U* test (non-normal distribution). Overall survival (OS) was defined as the time from diagnosis to death or the last follow-up for those still alive. The Kaplan-Meier method was used to determine survival rates, and the log-rank test was used to evaluate the differences between groups. Statistical analyses were conducted using R software (V 4.1.2), and differences were considered statistically significant when *P*-value < 0.05 (two-sided tests).

## Results

### Clinicopathological features

Of the 21 patients, 17 were male, and 4 were female, with a median age of 68 years (range 33–80). The maximum size of the carcinomas ranged from 2.5 cm to 14 cm, with a mean of 7.04 ± 2.89 cm. Histologically, 9 cases showed pure undifferentiated carcinoma, and the remaining 12 cases presented dedifferentiated carcinomas with visible differentiated adenocarcinoma components. Other significant findings included rhabdoid morphology in 8 cases, extensive necrosis in 7 cases, and vascular invasion in 13 cases. Microscopically, the undifferentiated carcinomas or undifferentiated components showed a sheet-like growth pattern with incohesive cells ranging from small round to large pleomorphic. Some carcinoma cells contained abundant eosinophilic cytoplasm showing rhabdoid morphology, whereas others contained reduced cytoplasm and increased nuclear/cytoplasmic ratio showing an immature appearance. The main clinicopathological features of the 21 cases are summarized in Table 2.

**Table 2:** Clinicopathologic features of 21 cases with undifferentiated/dedifferentiated gastric carcinoma

ID	Sex	Age	Final status	Follow-up (months)	Cell morphology	Differentiation type	Site	Size (cm)	TNM	Vascular invasion	Necrosis
1	M	68	AWD	74.50	Rhabdoid	Dedifferentiated	Gastric antrum	4.5	T4aN1M0 (IIIA)	Yes	No
2	M	61	DOD	5.53	Rhabdoid	Undifferentiated	Gastric body	7.5	T4aN3aM0 (IIIB)	Yes	No
3	M	65	AWD	61.47	Giant	Dedifferentiated	Gastric antrum	5	T4aN3aM0 (IIIB)	Yes	No
4	M	65	DOD	40.77	Small^a^	Dedifferentiated	Gastric antrum	2.8	T4bN0M0 (IIIA)	No	Yes
5	M	75	AWD	35.20	Undifferentiated	Dedifferentiated	Gastric body	12	T4aN0M0 (IIB)	Yes	No
6	M	58	DOD	22.30	Rhabdoid	Undifferentiated	Gastric antrum	6	T4aN3aM0 (IIIB)	Yes	No
7	M	70	DOD	1.63	Undifferentiated	Undifferentiated	Gastric antrum	4.5	T4aN2M1 (IV)	Yes	Yes
8	F	70	DOD	16.53	Rhabdoid	Undifferentiated	Gastric antrum	8	T4bN0M0 (IIIA)	Yes	No
9	M	70	AWD	31.20	Rhabdoid	Dedifferentiated	Gastric body	10	T4bN2M0 (IIIB)	Yes	Yes
10	F	55	DOD	11.57	Small^a^	Dedifferentiated	Gastric antrum	4	T2N2M0 (IIB)	No	No
11	M	70	AWD	24.17	Anaplastic	Undifferentiated	Gastric antrum	10	T4aN0M0 (IIB)	No	No
12	M	68	DOD	6.30	Anaplastic	Undifferentiated	Gastric body	5	T2N1M0 (IIA)	Yes	No
13	M	57	DOD	8.13	Undifferentiated	Undifferentiated	Gastric body	8	T4bN0M0 (IIIA)	Yes	No
14	M	66	DOD	18.93	Undifferentiated	Dedifferentiated	Gastric body	8	T3N0M0 (IIA)	No	Yes
15	M	75	DOD	9.13	Clear	Dedifferentiated	Gastric fundus	7	T4bN0M0 (IIIA)	No	Yes
16	M	61	DOD	13.07	Rhabdoid	Dedifferentiated	Gastric body	8	T4bN3aM0 (IIIC)	Yes	Yes
17	F	74	AWD	13.00	Medulla	Dedifferentiated	Gastric antrum	2.5	T2N0M0 (IB)	Yes	No
18	F	71	DOD	5.73	Undifferentiated	Undifferentiated	Gastric antrum	14	T3N1M0 (IIB)	No	Yes
19	M	80	AWD	5.03	Undifferentiated	Dedifferentiated	Gastric antrum	7	T4aN1M0 (IIIA)	No	No
20	M	54	DOD	4.00	Rhabdoid	Undifferentiated	Gastric antrum	6	T1N2M0 (IIA)	Yes	No
21	M	33	AWD	20.70	Rhabdoid	Dedifferentiated	Gastric antrum	8	T2N0M0 (IB)	No	No

*M* male, *F* female, *DOD* die of disease, *AWD* alive with disease

^a^Small: small cell size with high nucleocytoplasmic ratio

### Immunohistochemical findings

**Fig. 1:**
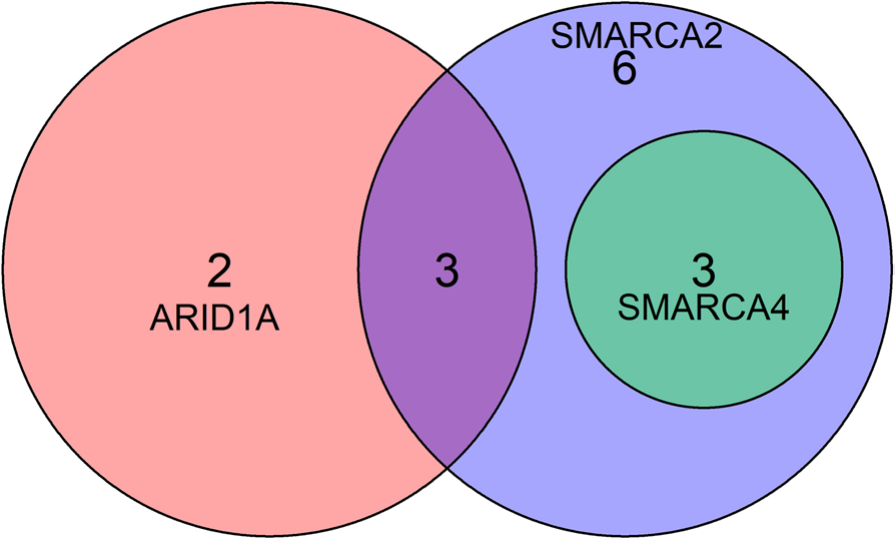
The Venn diagram showed concurrent loss of ARID1A, SMARCA2, and SMARCA4. The loss of SARMCA2 occurred most frequently and was often accompanied by simultaneous loss of other subunits. The 3 cases with SMARCA4 loss all showed concomitant SMARCA2 loss (i.e., SMARCA4 and SMARCA2 co-loss). Three cases showed ARID1A and SMARCA2 co-loss

All 21 enrolled cases showed the focal or variable intensity of CK-pan expression. We observed SMARCA2 loss in 12 cases (57.14%), ARID1A loss in 5 cases (23.81%), and SMARCA4 loss in 3 cases (14.29%); however, we did not find any case showing SMARCB1 or ARID1B loss. Taken together, 14 cases (66.67%) showed any one of the SWI/SNF complex subunits loss, including 3 cases with ARID1A and SMARCA2 co-loss and 3 cases with SMARCA4 and SMARCA2 co-loss (Fig. 1). Of note, the ARID1A and SMARCA2 co-loss group showed MMR protein deficiency and P53 wild type; conversely, the SMARCA4 and SMARCA2 co-loss group showed MMR protein proficiency and P53 mutation. MMR proteins deficiency was observed in 5 cases, of which all showed PMS2 loss, 3 showed MLH1 loss, 1 showed MSH6 loss, and none showed MSH2 loss. CSC phenotype was observed in 12 cases, including SOX2 positive expression in 11 cases and SALL4 positive expression in 5 cases. 11 cases showed P53 mutations, and 9 cases showed PD-L1 positive. Details of the immunohistochemical staining results are summarized in Table 3.

**Table 3:** Immunohistochemistry results of 21 cases with undifferentiated/dedifferentiated gastric carcinoma

ID	P53	PD-L1	ARID1A	ARID1B	SMARCA2	SMARCA4	SMARCB1	MSH2	MSH6	MLH1	PMS2	SOX2	SALL4
1	Mutate	Negative	Present	Present	Present	Present	Present	Present	Present	Present	Present	Positive	Positive
2	Mutate	Negative	Present	Present	Lost	Present	Present	Present	Present	Present	Present	Negative	Positive
3	Mutate	Positive	Present	Present	Present	Present	Present	Present	Present	Present	Present	Negative	Negative
4	Wild	Positive	Lost	Present	Present	Present	Present	Present	Present	Present	Present	Positive	Negative
5	Wild	Positive	Present	Present	Present	Present	Present	Present	Present	Lost	Lost	Negative	Negative
6	Mutate	Positive	Present	Present	Present	Present	Present	Present	Present	Present	Present	Negative	Negative
7	Wild	Negative	Present	Present	Lost	Present	Present	Present	Present	Present	Present	Positive	Negative
8	Wild	Positive	Lost	Present	Lost	Present	Present	Present	Present	Lost	Lost	Negative	Negative
9	Mutate	Positive	Present	Present	Present	Present	Present	Present	Present	Present	Present	Negative	Negative
10	Mutate	Negative	Present	Present	Lost	Lost	Present	Present	Present	Present	Present	Positive	Positive
11	Wild	Negative	Present	Present	Present	Present	Present	Present	Present	Present	Present	Negative	Negative
12	Mutate	Negative	Present	Present	Lost	Present	Present	Present	Present	Present	Present	Positive	Positive
13	Mutate	Positive	Present	Present	Lost	Lost	Present	Present	Present	Present	Present	Positive	Negative
14	Wild	Negative	Lost	Present	Lost	Present	Present	Present	Lost	Present	Lost	Positive	Negative
15	Mutate	Positive	Present	Present	Lost	Present	Present	Present	Present	Present	Present	Negative	Negative
16	Wild	Negative	Present	Present	Lost	Present	Present	Present	Present	Present	Present	Positive	Negative
17	Mutate	Negative	Present	Present	Present	Present	Present	Present	Present	Present	Present	Negative	Negative
18	Mutate	Negative	Present	Present	Lost	Lost	Present	Present	Present	Present	Present	Positive	Positive
19	Wild	Positive	Lost	Reduced	Present	Present	Present	Present	Present	Lost	Lost	Negative	Negative
20	Wild	Negative	Lost	Intact	Lost	Present	Present	Present	Present	Present	Lost	Positive	Negative
21	Wild	Negative	Present	Intact	Lost	n*: Lost s*: Present	Present	Present	Present	Present	Present	n*: Positive s*: Negative	Negative

n* nested, s* sheet-like

**Fig. 2:**
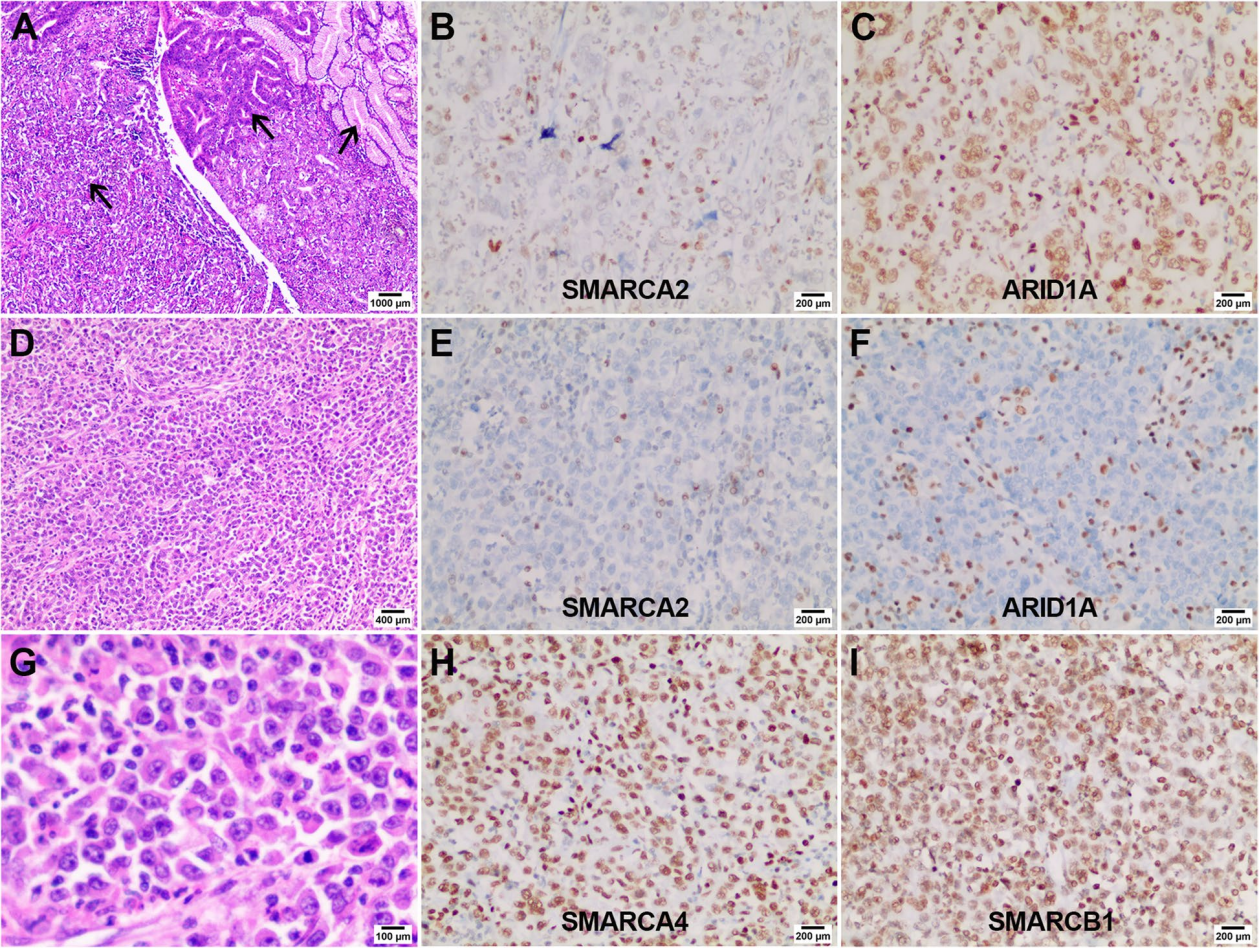
Representative images of dedifferentiated carcinoma and rhabdoid undifferentiated carcinoma. Case 17 was a typical dedifferentiated carcinoma and showed the coexistence of normal glands, well-differentiated adenocarcinoma components, and undifferentiated components at low magnification (**A**, arrows, × 40). The undifferentiated components showed reduced expression of SMARCA2 (**B**, × 200) and intact expression of ARID1A (**C**, × 200). Case 8 was a typical rhabdoid undifferentiated carcinoma and showed a non-cohesive sheet-like growth pattern at low magnification (**D**, × 100). The neoplastic cells presented polygonal or round with abundant eosinophilic cytoplasm, and the nuclei were large and vacuolated with prominent nucleoli at high magnification (**G**, ×400). SMARCA2 and ARID1A showed a complete loss, accompanied by strong staining of the surrounding lymphocytes (**E**, **F**, ×200), while SMARCA4 and SMARCB1 showed diffuse intense staining (**H**, **I**, ×200)

We found a typical dedifferentiated carcinoma (case 17) and a typical rhabdoid undifferentiated carcinoma (case 8) in this study (Fig. 2). H&E image of case 17 (A, × 40) showed the coexistence of normal glands, well-differentiated adenocarcinoma components, and undifferentiated components in which SMARCA2 expression was reduced (B, × 200), and ARID1A expression was intact (C, × 200). H&E image of the case 8 presented a non-cohesive sheet-like structure (D, ×100) and rhabdoid cell morphology (G, ×400) with loss of expression of SMARCA2 and ARID1A (E, F, ×200), and intact expression of SMARCA4 and SMARCB1 (H, I, ×200).

**Fig. 3:**
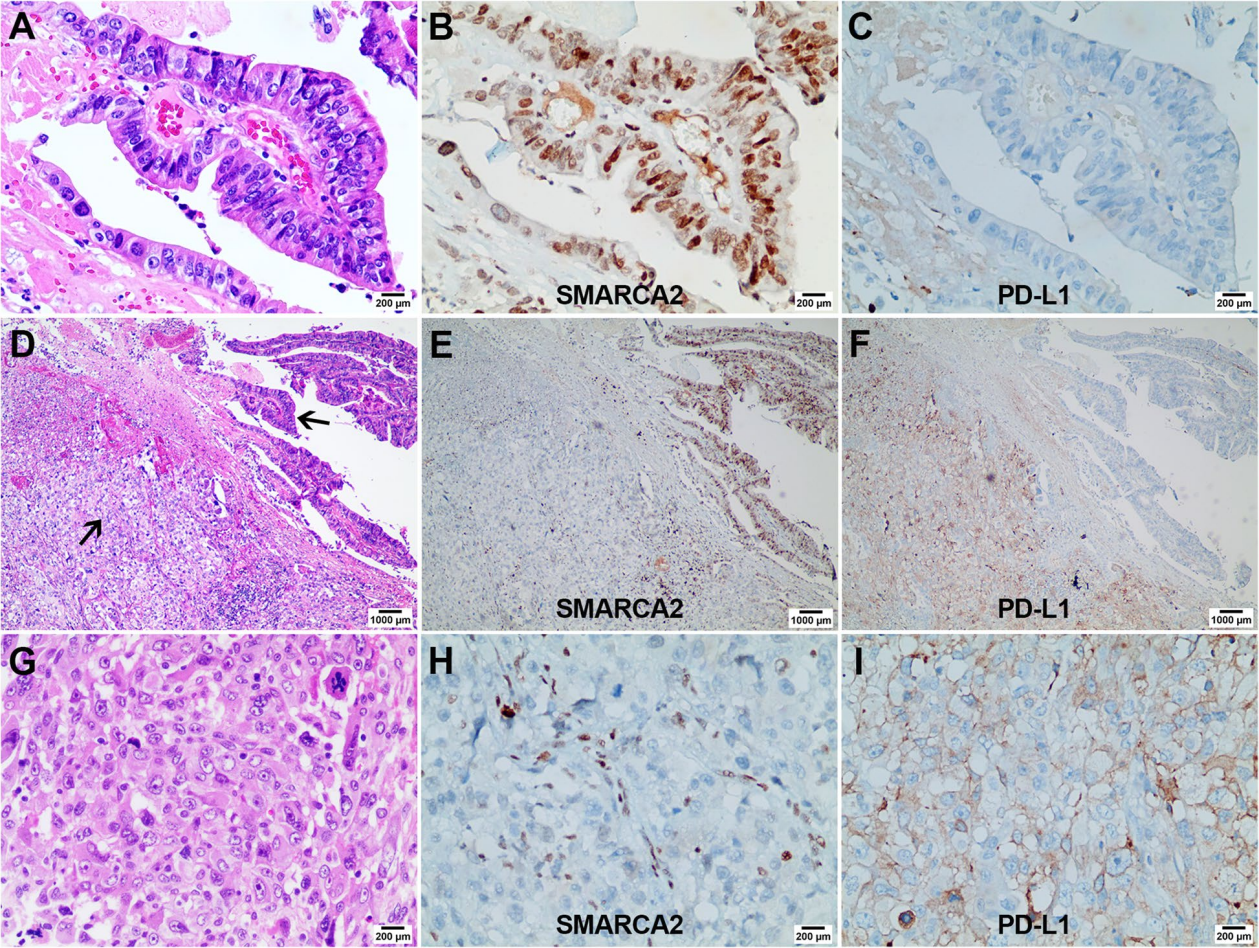
Heterogeneous expression pattern of SMARCA2 and PD-L1 in dedifferentiated carcinoma (case 15). Prominent adenocarcinoma components and undifferentiated components were observed in case 15 (**D**, arrows, ×40). The adenocarcinoma components showed a prominent glandular cavity (**A**, × 200), and the undifferentiated components showed neoplastic cells loosely arranged in a patchy pattern, a moderate amount of pale eosinophilic cytoplasm with a large nucleus; focal cytoplasm showed clear (**G**, ×200). In the adenocarcinoma components, SMARCA2 was intact (**B**, × 200), and PD-L1 was negative (**C**, × 200); while in the undifferentiated components, SMARCA2 showed complete loss (**H**, × 200), and PD-L1 showed diffused moderate positive expression (**I**, × 200)

We observed a heterogeneous expression of SMARCA2 and PD-L1 in case 15 (Fig. 3). In the adenocarcinoma components, SMARCA2 was intact (B, × 200) and PD-L1 was negative (C, × 200); conversely, in the undifferentiated components, SMARCA2 showed lost (H, × 200) and PD-L1 showed positive (I, × 200).

**Fig. 4:**
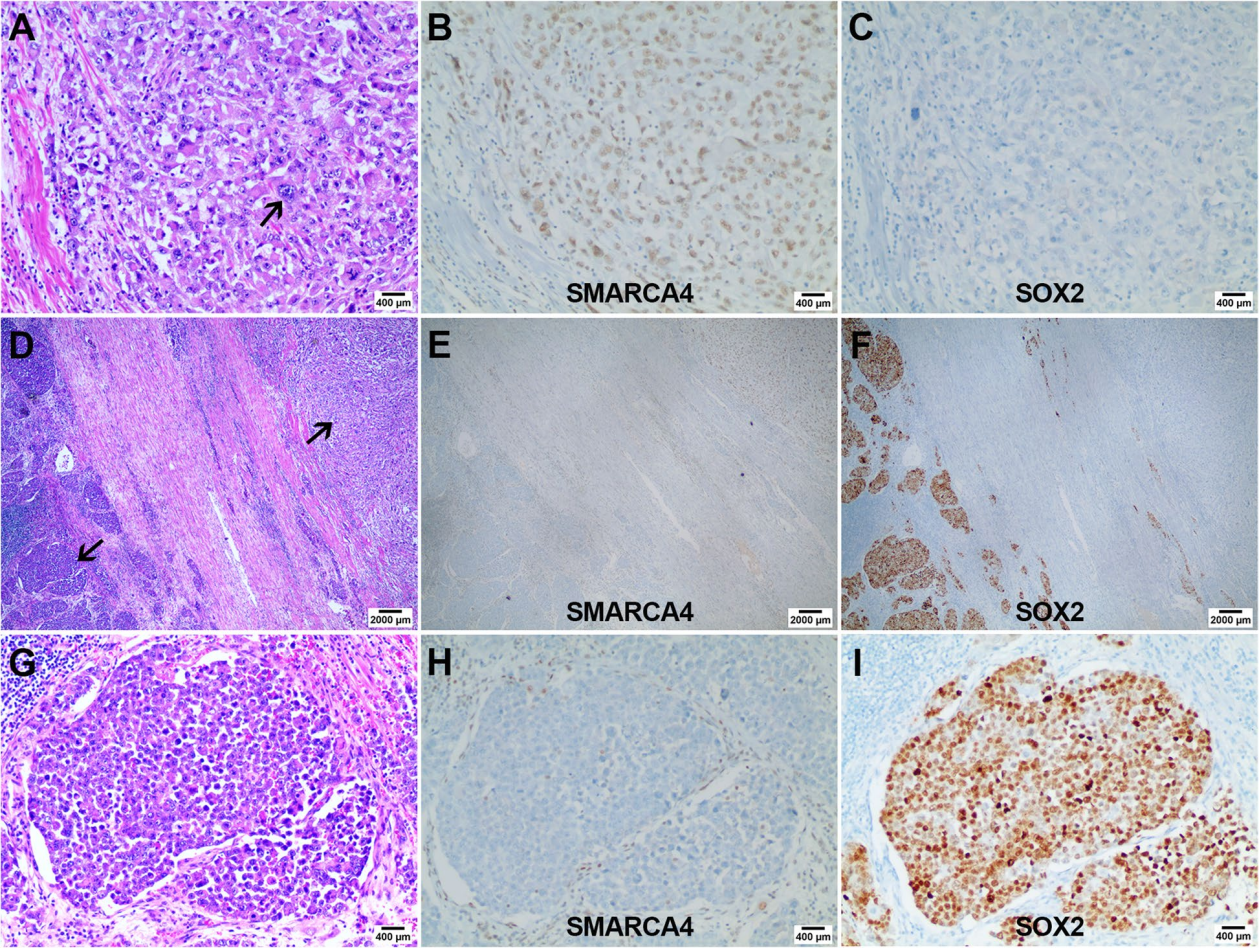
Heterogeneous expression pattern of SMARCA4 and SOX2 in undifferentiated carcinoma (case 21). Two kinds of histological structures were observed in case 21: nested and sheet-like structures (**D**, arrows, ×20), both of which were composed of undifferentiated neoplastic cells. In the sheet-like structure, neoplastic cells showed a pleomorphic appearance with abundant eosinophilic cytoplasm, and multinucleated tumor giant cells were occasionally observed (**A**, arrows, ×100). The nested structure was composed of monotonous small round neoplastic cells with poor cohesion, scant cytoplasm, and large nuclei (**G**, ×100). In sheet-like structure, SMARCA4 was intact (**B**, ×100) and SOX2 was negative (**C**, ×100), while in nested architecture, SMARCA4 showed complete loss (**H**, ×100) and SOX2 showed diffused intense positive expression (**I**, ×100)

We found two kinds of histological structures in case 21 (Fig. 4): nested and sheet-like structures (D×20, A, G×100), composed of undifferentiated neoplastic cells with distinct immunohistochemical expression profiles. In sheet-like structures, SMARCA4 was intact (B, ×100) and SOX2 was negative (C, ×100), while in nested architectures, SMARCA4 was lost (H, ×100) and SOX2 was positive (I, ×100).

### Correlation analysis

We performed correlation analyses between the expression status of SWI/SNF complex and clinicopathological characters and found that the CSC phenotype was more likely to appear in the SWI/SNF complex deficient group (*P* = 0.0158) (Table 4).

**Table 4:** Correlations of the SWI/SNF complex with clinicopathological characters

		SWI/SNF complex		
Variables	Total (*n* = 21)	Deficient (*n* = 14)	Intact (*n* = 7)	*P* value
Sex				1
Female	4 (19.05)	3 (21.43)	1 (14.29)	
Male	17 (80.95)	11 (78.57)	6 (85.71)	
Age				0.2941
Median (IQR)	68 (61,70)	65.5 (58,70)	70 (66.5,72)	
CellMorphology				1
NonRhabdoid	13 (61.9)	9 (64.29)	4 (57.14)	
Rhabdoid	8 (38.1)	5 (35.71)	3 (42.86)	
DifferentiationType				0.6424
Dedifferentiation	12 (57.14)	7 (50)	5 (71.43)	
Undifferentiated	9 (42.86)	7 (50)	2 (28.57)	
Site				1
Gastric antrum	13 (61.9)	8 (57.14)	5 (71.43)	
Gastric body	7 (33.33)	5 (35.71)	2 (28.57)	
Gastric fundus	1 (4.76)	1 (7.14)	0 (0)	
Size (cm)				0.9099
Mean (SD)	7.04 (2.89)	6.99 (2.66)	7.14 (3.52)	
T_Stage				0.6244
T1&T2	5 (23.81)	4 (28.57)	1 (14.29)	
T3&T4	16 (76.19)	10 (71.43)	6 (85.71)	
N_Stage				1
N-	9 (42.86)	6 (42.86)	3 (42.86)	
N+	12 (57.14)	8 (57.14)	4 (57.14)	
M_Stage				1
M0	20 (95.24)	13 (92.86)	7 (100)	
M1	1 (4.76)	1 (7.14)	0 (0)	
TNM_Stage				1
I&II	9 (42.86)	6 (42.86)	3 (42.86)	
III&IV	12 (57.14)	8 (57.14)	4 (57.14)	
VascularInvasion				0.1736
No	8 (38.1)	7 (50)	1 (14.29)	
Yes	13 (61.9)	7 (50)	6 (85.71)	
Necrosis				0.3371
No	14 (66.67)	8 (57.14)	6 (85.71)	
Yes	7 (33.33)	6 (42.86)	1 (14.29)	
P53				0.3615
Mutate	11 (52.38)	6 (42.86)	5 (71.43)	
Wild	10 (47.62)	8 (57.14)	2 (28.57)	
PD-L1				0.3972
Negative	12 (57.14)	9 (64.29)	3 (42.86)	
Positive	9 (42.86)	5 (35.71)	4 (57.14)	
MMR				0.6244
dMMR	5 (23.81)	4 (28.57)	1 (14.29)	
pMMR	16 (76.19)	10 (71.43)	6 (85.71)	
CSCphenotype				0.0158
Negative	9 (42.86)	3 (21.43)	6 (85.71)	
Positive	12 (57.14)	11 (78.57)	1 (14.29)	

*IQR* interquartile range, *SD* standard deviation, *MMR* mismatch repair proteins, *dMMR* MMR deficient, *pMMR* MMR proficient, *CSC* cancer stem cells

### Prognostic analysis

**Fig. 5:**
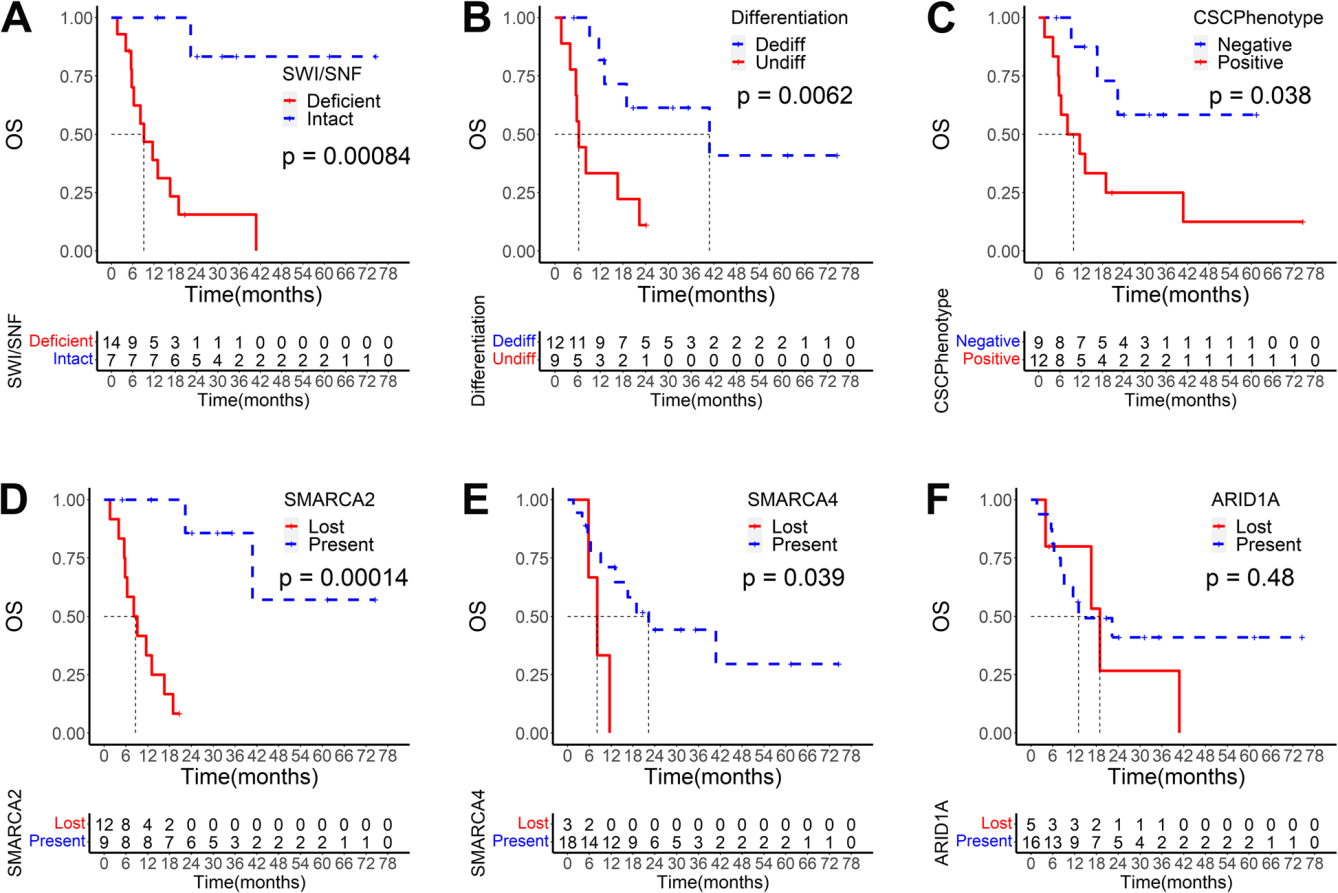
Kaplan-Meier survival curves for the undifferentiated and dedifferentiated gastric carcinoma. SWI/SNF complex deficient, undifferentiated status, CSC phenotype, SAMRCA2 loss, and SAMRCA4 loss were detrimental to overall survival (**A**, **B**, **C**, **D**, **E**). While ARID1A expression status had no impact on overall survival (**F**). OS, overall survival; Dediff, dedifferentiated; Undiff, undifferentiated; CSC, cancer stem cell

**Fig. 6:**
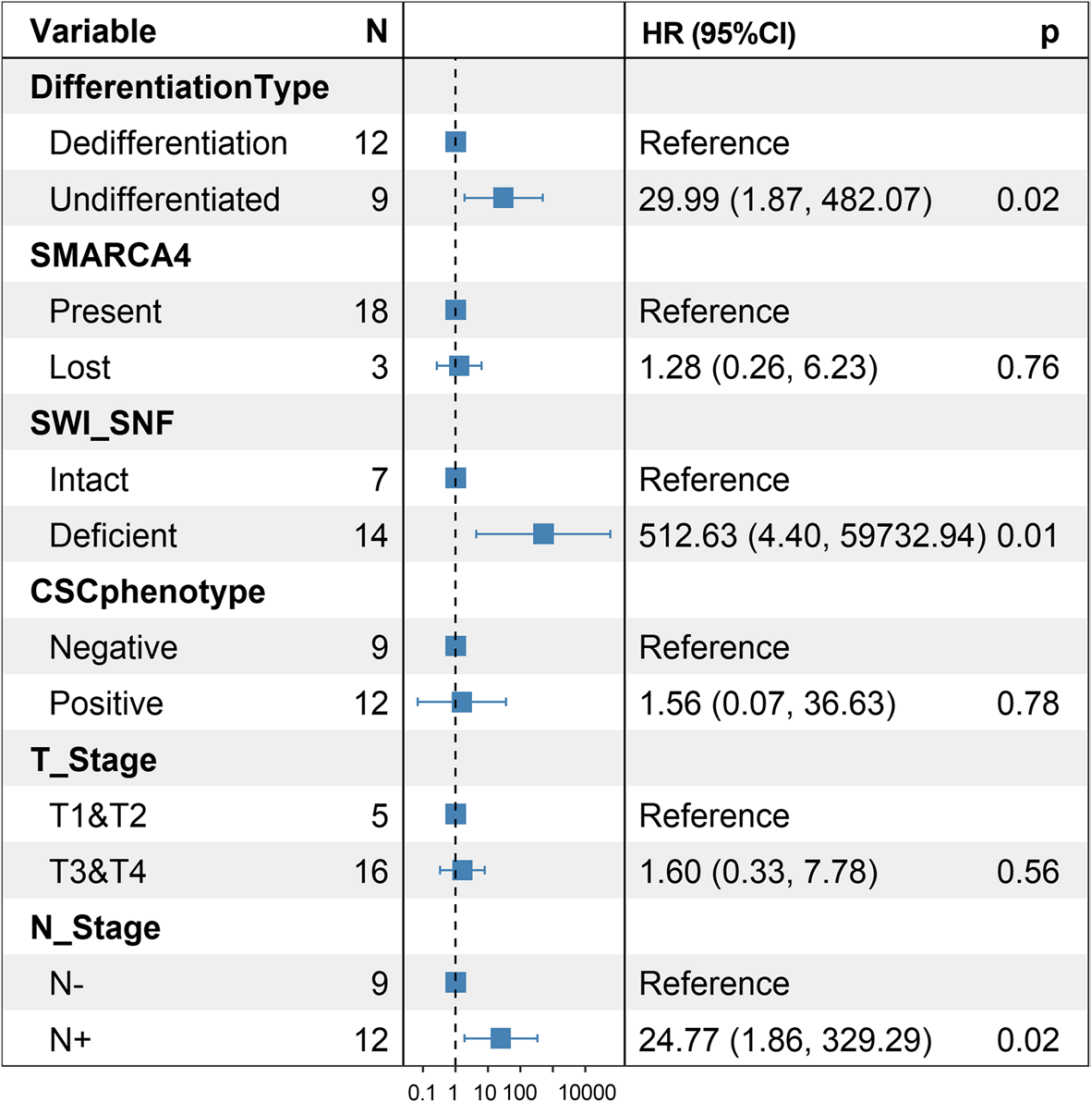
Forest plot of multivariate Cox regression analysis. Three variables independently associated with prognosis were screened: differentiation type, SWI/SNF complex, and N stage. HR, hazard ratio; CI, confidence interval; CSC, cancer stem cell

Survival analysis revealed that SWI/SNF complex deficiency, undifferentiated status, and CSC phenotype were associated with worse survival (*P* = 0.00084, 0.0062, and 0.038, respectively). For SWI/SNF complex subunits, both SMARCA2 and SMARCA4 showed worse survival in the lost group (*P* = 0.00014, and 0.039, respectively), whereas loss of ARID1A was not associated with survival (Fig. 5). We performed univariate Cox regression analysis and screened four variables with *P* < 0.1, including differentiation type, SMARCA4, SWI/SNF complex, and CSC phenotype (Table S1). In the subsequent multivariate Cox regression analysis, we included not only the above four variables but also the T stage and N stage, which were acknowledged to have an essential influence on prognosis. Finally, we obtained three variables independently associated with prognosis: differentiation type, SWI/SNF complex, and N stage (Fig. 6).

## Discussion

Currently, increasing evidence indicates that undifferentiated/dedifferentiated carcinoma is derived from the dedifferentiation of normal tissue. For instance, the same molecular aberration was found in the undifferentiated and corresponding endometrial components in dedifferentiated endometrial carcinoma [14]. Our current study also supports this notion, as we found a typical image (Fig. 2A) containing various components of normal glands, adenocarcinoma, and undifferentiated carcinoma. In this study, SMARCA2 exhibited the highest loss ratio (12/21, 57.14%), followed by ARID1A (5/21, 23.81%) and SMARCA4 (3/21, 14.29%). The loss ratio of the above three subunits in undifferentiated/dedifferentiated gastric carcinoma was significantly higher than that in ordinary gastric adenocarcinoma [8, 9], suggesting a correlation between the differentiation status and the loss of SWI/SNF complex subunits. Our current study also showed the CSC phenotype appeared more frequently in the SWI/SNF complex deficient group (Table 4). SWI/SNF inactivation can interfere with cell lineage-specific differentiation, resulting in cell arrest at an undifferentiated stage and acquisition of self-renewal capacity, and even cause progenitor cells to reverse to stem cells [15]. We infer that the SWI/SNF complex subunits loss is involved in the dedifferentiation process of tumors and further induces the CSC phenotype.

In this study, neither SMARCB1 nor ARID1B exhibited the lost expression pattern, and the loss of ARID1A did not correlate with survival. In contrast, the loss of SMARCA2 and SMARCA4 resulted in worse survival, implicating the essential role of the two subunits in undifferentiated/dedifferentiated gastric carcinoma. A pan-cancer study showed an opposing prognosis for SMARCA2 and SMARCA4 in several types of tumors [16]. We speculate that the two subunits do not function equally in different tumors despite their 75% structural homology [17].

The loss of SMARCA2 often underlies tumorigenesis caused by the loss of SMARCA4 [18]. In this study, the three cases with SMARCA4 loss identified in this study all showed synchronous SMARCA2 loss (i.e., SMARCA4 and SMARCA2 co-loss group), and three of the five cases with ARID1A loss displayed concomitant SMARCA2 loss (i.e., ARID1A and SMARCA2 co-loss group). The immunohistochemical expression patterns of MMR proteins and p53 differed between the two groups. ARID1A is involved in regulating MMR proteins [19], so the ARID1A and SMARCA2 co-loss group presented MMR protein deficiency, consistent with what has been reported in the literature [20]. The activity of p53 is dually regulated by SMARCA2 and SMARCA4 [21]; thus, p53 is mutated in SMARCA4 and SMARCA2 co-loss group, whereas p53 appears wild-type in ARID1A and SMARCA2 co-loss group due to the presence of the compensatory subunit SMARCA4.

Concurrent loss of SMARCA4 and SMARCA2 was identified in another study of undifferentiated gastrointestinal tract carcinomas [11] and have also been described in SCCOHT [22] and thoracic SMARCA4-DUT [23]. It is worth mentioning that thoracic SMARCA4-DUT shows strong expression of one or more stem cell markers, SOX2, CD34, or SALL4, in addition to the simultaneous loss of SMARCA4 and SMARCA2 [23, 24], which is consistent with our results that three cases with SMARCA4 loss showed concomitant loss of SMARCA2 and also SOX2 or SALL4 expression. Whether the new terminology “SMARCA4-DUT,” used by WHO in 2021 in thoracic tumors, applies equally to gastric carcinoma still needs more in-depth studies with larger samples. SCCOHT and thoracic SMARCA4-DUT share similarities in morphologic, immunophenotypic, and molecular features [22, 23], further demonstrating that SMARCA4-lost malignancies represent a unique subgroup of tumors with typical morphologic and immunohistochemical features [25].

Immunohistochemical loss of SMARCA2 and SMARCA4 can occur simultaneously or independently, and undifferentiated carcinomas with SMARCA2 loss without SMARCA4 loss have also been reported [26], suggesting that SMARCA2 loss was also involved in the process of tumor dedifferentiation [24]. In this study, SMARCA2 was the most frequently lost subunit, while it was not thoroughly investigated in undifferentiated carcinomas compared with SMARCA4. Many studies have demonstrated that the deficiency of SMARCA4 is closely associated with undifferentiated lesions. For example, SMARCA4 deficiency was the most common driver in small cell carcinoma of the ovary hypercalcemic type (SCCOHT) [22] and thoracic SMARCA4-deficient undifferentiated tumor (SMARCA4-DUT) [23], while the role of SMARCA2 in the development and progression of undifferentiated carcinomas has been neglected, and more studies are needed in the future.

SWI/SNF-deficient undifferentiated carcinoma is a rare type in histopathology that does not respond well to the therapeutic strategies used for ordinary gastric adenocarcinomas. Moreover, due to its rarity, large-scale clinical trials for a specific subunit are almost impossible, and there is no consensus regarding their effective treatment currently. The following options can be considered for the management of patients with SWI/SNF-deficient malignancies. In tumors with SMARCA4 loss, SMARCA2 serves as a promising therapeutic target, namely a synthetic lethal therapy targeting the ATPase domain or bromodomain of SMARCA2 [27]. Enhancer of zeste homolog 2 (EZH2) is the enzymatic catalytic subunit of polycomb repressive complex 2 (PRC2), and in tumors with SMARCA2 loss mediated by PRC2, EZH2 inhibitors show antitumor activity [28]. Studies have confirmed that the inactivation of the SWI/SNF complex can increase the sensitivity of tumors to immune checkpoint inhibitors (ICI), indicating ICI is also an important therapeutic option [29].

The limitations of this study were as follows. First, we enrolled only postoperative cases and excluded those who underwent needle biopsy since the latter’s tissue was too little to be eligible for this study. Second, we performed univariate and multivariate Cox regression analyses based on small sample size so that the conclusions may be biased. Finally, the reduced expression pattern of SWI/SNF complex subunits accounts for only a minority. We integrated them with the intact expression pattern, so the biological significance of reduced expression is unclear.

## Conclusion

In summary, we evaluated the expression of the SWI/SNF complex subunits in undifferentiated/dedifferentiated gastric carcinoma. We focused mainly on the correlation of the SWI/SNF complex with CSC phenotype and prognosis. We finally determined that the SWI/SNF complex deficiency was an independent prognostic factor for undifferentiated/dedifferentiated gastric carcinoma. Meanwhile, considering the poor prognosis of undifferentiated/dedifferentiated gastric carcinoma, it is recommended to carry out immunohistochemical examinations of SWI/SNF complex subunits, especially SMARCA2 and SMARCA4, further to implement stratification for precise and individualized treatment.

## Supplementary Information

**Additional file 1: Table S1.** Univariate and multivariate Cox regression analysis.

## Data Availability

The datasets for the current study are available from the corresponding author.

## Abbreviations

SWI/SNF: Switch/sucrose nonfermentable
ATPase: Adenosine triphosphatase
SMARCA4: SWI/SNF-related, matrix-associated, actin-dependent regulator of chromatin, subfamily A, member 4
SMARCA2: SWI/SNF-related, matrix-associated, actin-dependent regulator of chromatin, subfamily A, member 2
ARID1A: AT-rich interaction domain 1A
ARID1B: AT-rich interaction domain 1B
SMARCB1: SWI/SNF-related, matrix-associated, actin-dependent regulator of chromatin, subfamily B, member 1
SOX2: SRY-box transcription factor 2
SALL4: Palt-like transcription factor 4
MLH1: MutL homolog 1
MSH2: MutS homolog 2
MSH6: MutS homolog 6
PMS2: PMS1 homolog, mismatch repair system component 2
EBER-ISH: Epstein-Barr virus (EBV)-encoded small RNA in situ hybridization
MMR: Mismatch repair
dMMR: MMR-deficient
pMMR: MMR-proficient
H&E: Hematoxylin and eosin
FFPE: Formalin-fixed and paraffin-embedded
OS: Overall survival
CSC: Cancer stem cells
SCCOHT: Small cell carcinoma of the ovary hypercalcemic type
SMARCA4-DUT: SMARCA4-deficient undifferentiated tumor
SDUS: SMARCA4-deficient uterine sarcomas
EZH2: Enhancer of zeste homolog 2
PRC2: Polycomb repressive complex 2
ICI: Immune checkpoint inhibitors
